# Transcriptome Analysis of BAFF/BAFF-R System in Murine Nephrotoxic Serum Nephritis

**DOI:** 10.3390/ijms25105415

**Published:** 2024-05-16

**Authors:** Tamara Möckel, Sebastian Boegel, Andreas Schwarting

**Affiliations:** 1 Division of Rheumatology and Clinical Immunology, Department of Internal Medicine I, University Medical Center of the Johannes Gutenberg University, 55131 Mainz, Germany; tamara.moeckel@unimedizin-mainz.de (T.M.); seb.boegel@gmail.com (S.B.); 2 Center for Rheumatic Disease Rhineland-Palatinate GmbH, 55543 Bad Kreuznach, Germany

**Keywords:** BAFF, BAFF-R, NTS, NTN, CKD, GN

## Abstract

Chronic kidney disease (CKD) is an emerging cause for morbidity and mortality worldwide. Acute kidney injury (AKI) can transition to CKD and finally to end-stage renal disease (ESRD). Targeted treatment is still unavailable. NF-*κ*B signaling is associated with CKD and activated by B cell activating factor (BAFF) via BAFF-R binding. In turn, renal tubular epithelial cells (TECs) are critical for the progression of fibrosis and producing BAFF. Therefore, the direct involvement of the BAFF/BAFF-R system to the pathogenesis of CKD is conceivable. We performed non-accelerated nephrotoxic serum nephritis (NTN) as the CKD model in BAFF KO (B6.129S2-*Tnfsf13b^tm1Msc^*/J), BAFF-R KO (B6(Cg)-*Tnfrsf13c^tm1Mass^*/J) and wildtype (C57BL/6J) mice to analyze the BAFF/BAFF-R system in anti-glomerular basement membrane (GBM) disease using high throughput RNA sequencing. We found that BAFF signaling is directly involved in the upregulation of collagen III as BAFF ko mice showed a reduced expression. However, these effects were not mediated via BAFF-R. We identified several upregulated genes that could explain the effects of BAFF in chronic kidney injury such as *Txnip*, *Gpx3*, *Igfbp7*, *Ccn2*, *Kap*, *Umod* and *Ren1*. Thus, we conclude that targeted treatment with anti-BAFF drugs such as belimumab may reduce chronic kidney damage. Furthermore, upregulated genes may be useful prognostic CKD biomarkers.

## 1. Introduction

In general, kidney injuries can be classified into the following two types: acute kidney injury (AKI) and chronic kidney injury (CKD). AKI is associated with bacterial infection, sepsis or ischemia/reperfusion (I/R) injury, whereas CKD is primarily caused by diabetic complications, hypertension, obesity or autoimmunity [1]. Thus, AKI and CKD differ in their initiating events. CKD affects more than 10% of the global population in developed countries [2] and is an emerging cause for morbidity and mortality worldwide as targeted treatment is still unavailable [3,4,5]. Currently, CKD is defined as abnormalities of kidney function or structure, which is present for more than three months [6] and an estimated glomerular filtration rate (eGFR) less than 60 mL/min/1.73 m^2^ on at least two occasions 90 days apart [7]. According to the GFR level, the severity of CKD is classified into five stages. In the case of a transition to end-stage renal disease (ESRD), patients require renal replacement therapy in the form of lifelong hemodialysis or renal transplantation.

Glomerulonephritis (GN) includes immune-mediated diseases and is characterized by damage to the glomerular compartment of the renal nephrons [8]. Acute GN accompanies hypertension, proteinuria and hematuria, whereas GN with podocyte injury causes nephrotic syndrome with massive proteinuria [9]. Both can lead to CKD and irreversible kidney failure [10,11]. In general, GN can be divided into the following five categories according to Anders et al.: infection-related, autoimmune, alloimmune, autoinflammatory and monoclonal gammopathy-related GN [9]. Autoimmune GN is characterized by the response of the adaptive immune system against several self-antigens, which can be expressed in the kidney itself or systemically. Lupus nephritis (LN) is a form of autoimmune GN, characterized by the loss of tolerance to chromatin components and other self-antigens [12]. Although several drugs are currently under evaluation in clinical trials, overview given in [9], treatment options are limited and still focused on glucocorticoids to control activity, which are nonspecific and show side effects.

In the context of the progression of renal fibrosis in kidney diseases, the focus was initially on (myo)fibroblasts. In the meantime, several studies showed that tubular epithelial cells (TECs) also play an important role [13]. Furthermore, it is assumed that TECs manage the progression from acute to chronic renal disease [14]. It is shown that in case of mild and transient injury, TECs manage the regeneration and therefore regain the kidney function [15,16]. However, TECs undergo maladaptive repair and thus exacerbate renal fibrosis when injury is severe and persists [16,17,18]. An overview of the intracellular pathways involved in TECs upon injury and that foster renal fibrosis are summarized in the review of Qi and Yang [19]. There is evidence suggesting that the severity and frequency of TECs injury defines whether repair mechanisms lead to recovery or progression of fibrosis [20]. Nevertheless, the progression of renal fibrosis in CKD is not fully understood so far.

NF-*κ*B signaling is activated by an increase in inflammatory cytokines and associated with chronic diseases like CKD [21]. In the case of type 2 diabetic nephropathy, the NF-*κ*B pathway is activated in renal TECs and significantly correlates with interstitial inflammation and proteinuria [22]. In general, an upregulation of NF-*κ*B is described in many different renal diseases like IgA nephropathy, crescentic GN, LN, minimal change disease and membranous nephropathy [23]. Suppressing NF-*κ*B with the inhibitor JSH-23 reduces the production of inflammatory cytokines and alleviates renal inflammation [24,25,26].

The B cell activating factor BAFF activates the non-canonical as well as canonical NF-*κ*B pathway via BAFF-R binding [27,28,29] and promotes the activation, differentiation and survival of B cells [30,31]. Therefore, BAFF is a key therapeutic target for several autoimmune diseases [32,33]. Blocking BAFF reduced the glucocorticoid dosage and prevented organ damage in SLE patients [34]. Furthermore, BAFF IgG complexes correlated with disease activity in SLE patients [35]. BAFF plays a role in LN by inducing renal tertiary lymphoid structures (TLSs) and regulating the position of T cells within glomeruli [36]. Renal pathology is associated with elevated BAFF production from cells within kidneys, the renal infiltration of immune cells, and the development of TLSs and glomerular deposits of IgG/C3. Furthermore, patients with proliferative LN can be classified into three different groups; one is the BAFF-dominant group [37]. BAFF and its receptors showed differential expression patterns according to LN classes [38]. Pathological class II of LN kidney biopsies showed tubulointerstitial BAFF and BCMA expression, but no expression of BAFF-R or TACI. For class III biopsies, tubulointerstitial BAFF, BCMA and TACI expression was detected as well as the glomerular expression of BAFF and TACI. The class IV expression pattern of BAFF and TACI was similar to class III, but glomerular BAFF and TACI expression was higher and BAFF-R was expressed interstitial. In the case of class V biopsies, BAFF and BCMA were expressed interstitial as well as glomerular, while TACI was expressed only glomerular and no BAFF-R expression was detected [38]. For IgA nephropathy, it is known that BAFF enhances the expression of fibroblast factors in kidneys by activating the TRAF6/NF-*κ*B signaling pathway [39]. BAFF activates B cells through the NF-*κ*B signaling pathway to secrete excess IgA1, which leads to IgA nephropathy-like alterations in mouse kidneys [40]. B cell activation and elevated BAFF levels are present in patients with IgA nephropathy [41,42]. There is considerable evidence that BAFF contributes to the pathogenesis of glomerulonephritis [43]. In patients with glomerulonephritis, BAFF levels are higher, and receptors are increased in the tubulointerstitial area [44].

Renal TECs are critical for the progression of renal fibrosis and produce the cytokine BAFF [45]. Taking into account that BAFF also has regulatory functions, it is conceivable that the BAFF/BAFF-R system is directly involved in the pathogenesis of chronic kidney diseases like GN. With regard to B cell-directed therapies, belimumab, a monoclonal antibody (mAb) against the B cell activating factor (BAFF), was approved by the FDA firstly as a biologic for adult (2011) and pediatric (2019) SLE patients [46,47,48] and additionally for adult (2020) and pediatric (2022) LN patients [47].

Against this background, our study was focused on investigating the cytokine BAFF and its receptors during renal fibrosis processes in the CKD model of nephrotoxic serum nephritis (NTN). The underlying model of this study is based on the non-accelerated murine nephrotoxic serum nephritis (NTN) model, which was developed as an acute model of GN [49,50,51,52] and evaluated as a CKD model by Ougaard et al. [53]. Pathogenesis is initiated by anti-glomerular IgGs that impair the glomerular filtration barrier and induce proteinuria and inflammation. The advantage of our study is the fact that investigations of the BAFF/BAFF-R system in autoimmune GN were carried out in mice not prone to autoimmunity, which enabled us to focus exclusively on the initiated mechanism of GN.

By using high throughput sequencing and following bioinformatical analyses with well-established in silico methods, we tried to identify among the multitude of genes those that have the greatest potential to be evaluated as useful biomarkers in further studies. Furthermore, we generated hypotheses concerning conspicuous genes in fibrotic kidneys relevant for CKD progression and the influence of BAFF as well as BAFF-R knockout for fibrosis progression in kidney disease.

## 2. Results

**Figure 1:**
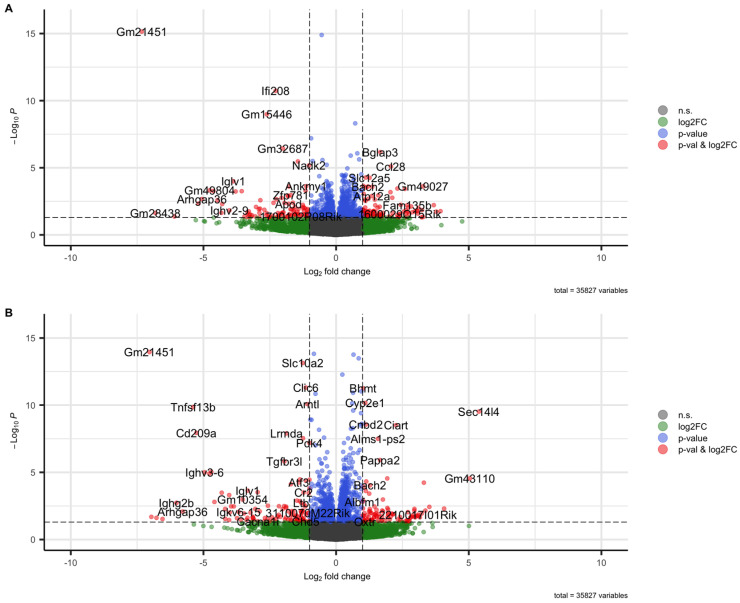
Volcano plots visualizing differentially regulated genes in kidneys 21 days after administration of nephrotoxic serum. (**A**) BAFF-R KO (B6(Cg)-*Tnfrsf13c^tm1Mass^*/J) vs. wildtype (C57BL/6J). (**B**) BAFF KO (B6.129S2-*Tnfsf13b^tm1Msc^*/J) vs. wildtype (C57BL/6J) strain.

In order to evaluate the role of the BAFF/BAFF-R system during the chronic phase of renal fibrosis, a murine CKD model was performed. Therefore, the renal transcriptome of BAFF and BAFF-R knockout (ko) mice was analyzed and compared to mice of a wildtype (wt) strain, 21 days after a single intravenous tail injection of nephrotoxic serum (NTS). A total of 35827 genes were analyzed, thereby 51 up-(log2FC ≥ 1, *p* < 0.05) and 53 downregulated (log2FC ≤ −1, *p* < 0.05) genes detected in BAFF ko kidneys versus wildtype and 77 up-(log2FC ≥ 1, *p* < 0.05) and 82 downregulated (log2FC ≤ −1, *p* < 0.05) genes in BAFF-R ko kidneys compared to the wildtype (Figure 1).

**Figure 2:**
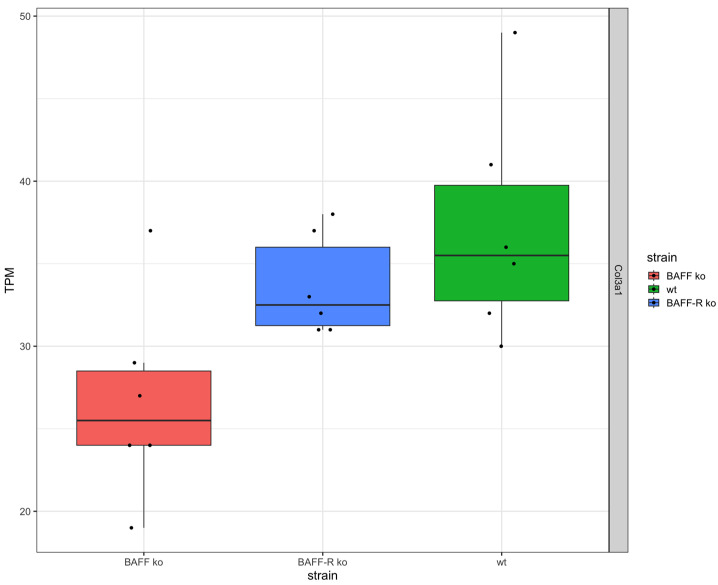
Collagen III expression in kidneys 21 days after administration of nephrotoxic serum in BAFF KO (B6.129S2-Tnfsf13b^tm1Msc^/J), BAFF-R KO (B6(Cg)-Tnfrsf13c^tm1Mass^/J) and wildtype (C57BL/6J) strain visualized as transcripts per million (TPM). *Col3a1*: Collagen Type III Alpha 1. ko: knockout, wt: wildtype. Each point shows one sample.

Fibrosis marker collagen III (*Col3a1*) was expressed in the kidneys of all three strains (Figure 2). The expression level in the BAFF-R ko (median: 32.5 TPM) and wildtype (median: 35.5 TPM) was similar and higher in comparison to the BAFF ko kidneys (median: 25.5 TPM).

**Figure 3:**
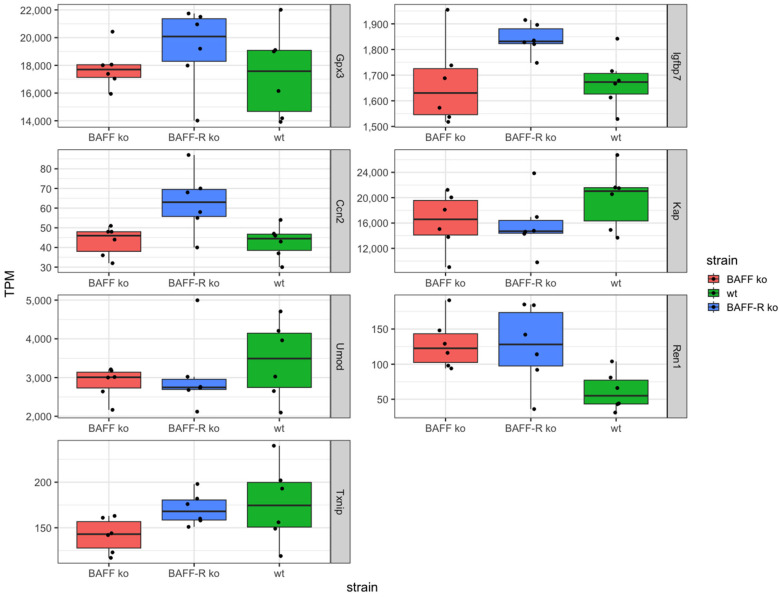
Identified genes in kidneys 21 days after administration of nephrotoxic serum in BAFF KO (B6.129S2-Tnfsf13b^tm1Msc^/J), BAFF-R KO (B6(Cg)-Tnfrsf13c^tm1Mass^/J) and wildtype (C57BL/6J) strain, visualized as transcripts per million (TPM). *Gpx3*: Glutathione Peroxidase 3, *Igfbp7*: Insulin-Like Growth Factor Binding Protein 7, *Ccn2*: Cellular Communication Network Factor 2, *Kap*: Kidney androgen regulated protein, *Umod*: Uromodulin, *Ren1*: Renin, *Txnip*: Thioredoxin Interacting Protein. ko: knockout, wt: wildtype. Each point shows one sample.

Furthermore, based on the limited knowledge of triggers and predictive biomarkers concerning the turnover of AKI to CKD, sequencing should provide prominently regulated genes as potential markers. Thereby, we identified the following seven genes: *Gpx3*, *Igfbp7*, *Ccn2*, *Kap*, *Umod*, *Ren1* and *Txnip* (Figure 3).

The identified genes *Gpx3*, *Igfbp7* and *Ccn2* showed similar expression levels in BAFF ko (*Gpx3* median: 17698 TPM, *Igfbp7* median: 1630, *Ccn2* median: 46 TPM) and wildtype (*Gpx3* median: 17578 TPM, *Igfbp7* median: 1673, *Ccn2* median: 44.5 TPM) and an upregulation in BAFF-R ko (*Gpx3* median: 20080 TPM, *Igfbp7* median: 1832, *Ccn2* median: 63 TPM) as compared to the other two strains (Figure 3).

In contrast, the two genes *Kap* and *Umod* showed higher expression levels in wildtype kidneys (*Kap* median: 21030 TPM, *Umod*: 3493 TPM) compared to BAFF (*Kap* median: 16578 TPM, *Umod*: 3009 TPM) and BAFF-R (*Kap* median: 14703 TPM, *Umod*: 2747 TPM) knockout strains (Figure 3). Identified gene *Ren1* exhibited the opposite expression pattern with a lower median expression level in wildtype (median: 55 TPM) compared to BAFF (median: 122 TPM) and BAFF-R (128 TPM) ko mice (Figure 3).

The *Txnip* expression level was the lowest in BAFF ko kidneys (median: 143 TPM) and similar in BAFF-R ko (median: 168 TPM) and wildtype (median: 174 TPM) mice (Figure 3).

**Figure 4:**
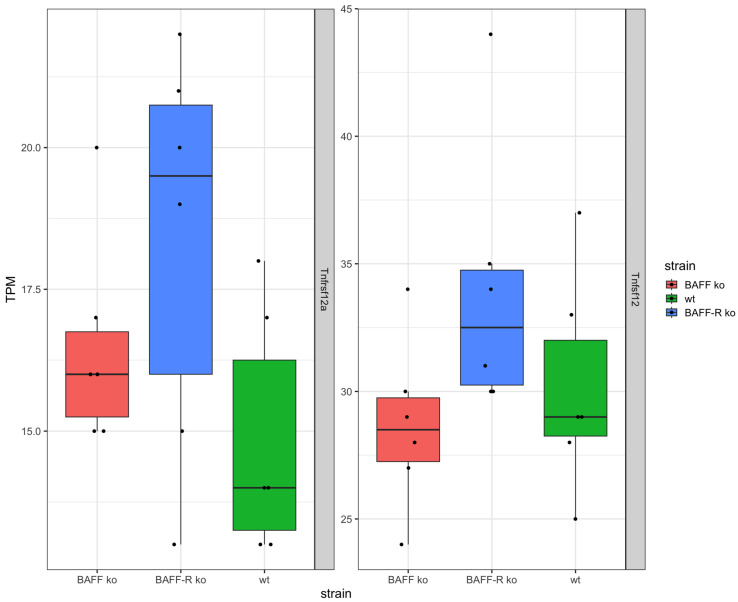
Analyzed genes (TWEAK/TWEAK receptor system) of kidneys 21 days after the administration of nephrotoxic serum in BAFF KO (B6.129S2-Tnfsf13b^tm1Msc^/J), BAFF-R KO (B6(Cg)-Tnfrsf13c^tm1Mass^/J) and wildtype (C57BL/6J) strain, visualized as transcripts per million (TPM). *Tnfsf12*: TWEAK, *Tnfrsf12a*: TWEAK receptor. ko: knockout, wt: wildtype. Each point shows one sample.

In addition, the TNF superfamily member TWEAK (*Tnfsf12*) and its receptor (*Tnfrsf12a*) were expressed in the kidneys of the three mouse strains (Figure 4). Thereby, the expression of TWEAK and its receptor were similar in BAFF ko (*Tnfsf12* median: 28.5 TPM, *Tnfrsf12a* median: 12 TPM), BAFF-R ko (*Tnfsf12* median: 32.5 TPM, *Tnfrsf12a* median: 19.5 TPM) and wildtype kidneys (*Tnfsf12* median: 29 TPM, *Tnfrsf12a* median: 14 TPM). However, the expression of TWEAK was higher compared to the expression level of the receptor in all three strains.

## 3. Discussion

In our previous work [54], we investigated the BAFF/BAFF-R system during acute kidney injury (AKI) by using the ischemia/reperfusion (I/R) model. In the present study, we now focus on the cytokine BAFF and its receptors during renal fibrosis processes in the chronic kidney disease (CKD) model of nephrotoxic serum nephritis (NTN). The transcriptome of BAFF ko, BAFF-R ko and wildtype kidneys was analyzed 21 days after the administration of nephrotoxic serum with regard to differentially expressed genes.

Ougaard et al. have recently reported that NTN originally associated with acute kidney glomerulonephritis can serve as an excellent model for CKD [53]. An advantage of our study design was the fact that used strains were not prone to autoimmunity, which enabled us to discover the impact of the BAFF/BAFF-R system during autoimmune GN without genetic determined autoimmune influence.

As expected, all strains showed an expression of collagen III (*Col3a1*) as evidence for the development of renal fibrosis in the chronic phase of kidney injury. Interestingly, the expression level of *Col3a1* was lower in BAFF ko kidneys compared to BAFF-R ko and wildtype kidneys. This finding indicates that the knockout of the cytokine BAFF seemed to improve chronic kidney damage by reducing the development of renal fibrosis. It is known that the inhibition of BAFF attenuates fibrosis in scleroderma [55]. In systemic sclerosis, BAFF promotes collagen and profibrotic marker expression by dermal fibroblasts [56]. A study of Thapa et al. in 2020 showed that targeting BAFF attenuates autoantibody production which is associated with cholestatic liver disease and that BAFF is a potential target for hepatic fibrosis [57]. Furthermore, it is known that B cells promote myocardial collagen type I and III expression [58].

The same expression pattern was detected for Thioredoxin Interacting Protein (*Txnip*). This gene showed the lowest gene expression in kidneys of the BAFF ko strain, whereas the expression in the BAFF-R ko and wildtype was similar. Txnip is a regulator of cellular redox signaling and thus protects cells from oxidative stress. The expression of *Txnip* is upregulated in kidneys after unilateral ureteral obstruction (UUO) and the knockout mice were protected [59]. Therefore, Txnip seems to play a role in renal fibrosis and progression to CKD. Txnip expression significantly increased in human proteinuric kidney diseases like focal segmental glomerulosclerosis (FSGS), membranous nephropathy (MN) and diabetic nephropathy (DN) [60]. Its inhibition by CHOP deletion suppresses NLRP3 inflammasome activation and p-ASK1-dependent mitochondrial apoptosis, which decreased albuminuria and improved renal function in nephrotic syndrome (NS) [60]. Furthermore, UUO-induced renal inflammation is suppressed by inhibiting the activation of NF-*κ*B and NLRP3 inflammasome [59]. Txnip interacts with STAT3 and promotes STAT3 signaling pathway-activated profibrotic response [61]. The upregulation of Txnip is observed and implicated in pathological pathways in vivo and in vitro in NS, UUO-induced renal fibrosis, aging-related renal fibrosis and the DN model, as well in human proteinuric kidney disease. The genetic deletion of *Txnip* resulted in reduced oxidative stress, renal fibrosis and extracellular matrix accumulation, podocyte injury and inflammation [59]. The lower *Txnip* expression in BAFF ko kidneys may imply that the absence of BAFF mediated protective effects. Interestingly, the higher expression in BAFF-R ko suggests that BAFF signaling worsened the outcome, but that this signaling is not mediated via BAFF-R. The fact that *Txnip* expression in wildtype kidneys was similar to its expression pattern in BAFF-R ko strain kidneys supports this hypothesis. Furthermore, we have recently observed similar effects in BAFF and BAFF-R ko mice in a model of acute kidney injury [54].

In addition, even *Gpx3*, *Igfbp7* and *Ccn2* showed this expression pattern with a higher expression level in BAFF-R ko compared to BAFF ko kidneys. Glutathione Peroxidase 3 (Gpx3) is a selenoprotein and catalyzes the reduction of organic hydroperoxides and hydrogen peroxide via glutathione, therefore protecting cells against oxidative damage. It is primarily secreted by renal TECs of the proximal section [62,63] and binds in vitro as well as in vivo to the basement membrane of proximal and distal TECs in the renal cortex of mice [64]. A related pathway is the cellular response to stimuli and the activity of *Gpx3*, which is associated with CKD. In CKD patients, the level of Gpx3 is reduced compared to healthy individuals [62,65]. Another study detected *Gpx3* upregulation after an I/R injury of the kidney in mice [66]. Therefore, a higher expression of *Gpx3* in BAFF-R ko kidneys may imply that the induced renal damage was larger and led to a higher *Gpx3* expression in order to compensate. However, expression level alone provides no information about the function and activity at protein level. Insulin-Like Growth Factor Binding Protein 7 (Igfbp7) stimulates cell adhesion and is related to cellular response to stimuli pathway as well. Besides Timp-2, Igfbp7 was the first urinary biomarker for risk stratification with regard to developing AKI, which was approved in 2014 by the FDA [67,68]. The expression level of *Igfbp7* was high in all three strains, but highest in BAFF-R ko. Again, this could mean that damage in this strain was larger compared to the BAFF ko and wildtype strain, which led to the higher *Igfbp7* expression. The Cellular Communication Network Factor 2 (Ccn2) is a component of the extracellular matrix (ECM), involved in cellular signaling and related to renal fibrosis [69,70,71]. Therefore, Ccn2 is discussed as a fibrotic biomarker [72,73]. Mouse studies revealed that Ccn2 binds to renal TECs and thereby contributes to renal damage [74]. Against this background, the expression pattern of *Ccn2* in our study would mean that damage in BAFF-R ko kidneys induced by the administration of NTS was higher compared to BAFF ko and wildtype kidneys.

Our finding of lower expression levels in BAFF ko kidneys which indicates a protective effect with regard to renal fibrosis is in line with the literature. In the case of IgA nephropathy, BAFF enhances the expression of fibroblast factors by activating the TRAF6/NF-*κ*B signaling pathway in a rat model [39]. It is also known that BAFF activates B cells through the NF-*κ*B signaling pathway in patients with IgA nephropathy [40] and that B cell activation and elevated BAFF levels are present in these patients [41,42]. Plasma levels of BAFF positively correlated with the Katafuchi score. In GN patients, BAFF levels are higher and receptors increased in tubulointerstitial area [44]. BAFF exerted a proliferative effect on human mesangial cells in vitro through BAFF-R; therefore, BAFF may contribute to the pathogenesis of glomerulonephritis [43]. In addition, anti-BAFF therapy with belimumab was recently approved for the use in LN due to its beneficial effects [75].

However, in our study, we did not find any evidence of BAFF-R (*Tnfrsf13c*) or BCMA (*Tnfrsf17*) expression, as there were no reads mapping to these transcripts in the majority of mice over all conditions. In contrast, the expression of TACI (*Tnfrsf13b*) cannot be excluded (Figure S1). Nevertheless, in regard to TNF superfamily members, TWEAK (*Tnfsf12*) was detected on RNA level as well its receptor (*Tnfrsf12a*). It is known that the TWEAK/TWEAK receptor pathway plays a role in the pathogenesis of nephritis [76]. Anti-TWEAK treatment with monoclonal antibodies (mAb) attenuated glomerular and tubular damage and tubulointerstitial fibrosis. The TWEAK/Fn14 pathway promotes mesangial cell proliferation, vascular cell activation and renal cell death [76]. The anti-TWEAK mAb administration into WT mice in the NTN model ameliorated proteinuria and improved kidney histology, and decreased glomerular Ig deposition, macrophage infiltrates and tubulointerstitial fibrosis [76]. Fn14 is induced in injured and diseased tissue, expressed by mesangial cells, podocytes, endothelial cells and tubular cells [77,78,79,80]. TWEAK can promote tubular cell death [81,82] and protein levels elevated in lupus patients with active nephritis, and can increase during nephritic flares [83,84]. TWEAK and Fn14 mRNA is upregulated in glomerular and tubular compartments in human LN [85]. For one thing, autophagy in renal fibrosis can degrade unnecessary or dysfunctional components and therefore prevent cell apoptosis. Apart from that, it is also possible that damaged TECs do not undergo apoptosis and thus survive via autophagy. Thereby, TECs undergo maladaptive repair, phenotype changes and TECs, producing proinflammatory and profibrotic cytokines. Finally, renal fibrosis aggravates [86]. Furthermore, the senescence of TEC in CKD is induced by hypertension, diabetes or IgA nephropathy [16]. The characteristics of senescence are cell cycle arrest at the G2/M phase and the secretion of proinflammatory and profibrotic factors [16,17,18], which links tubular cell senescence with renal fibrosis. For the above-mentioned reasons, it can be assumed that besides the cytokine BAFF, even TWEAK and TWEAK receptor signaling is related to the chronic phase of renal disease. The chronological order of these events has to be further investigated.

In addition, three further genes—*Kap*, *Umod* and *Ren1*—were detected in our analyses. The expression pattern of Kidney androgen regulated protein (*Kap*) and Uromodulin (*Umod*) was similar to each other. Higher gene expression was detected in the wildtype strain, whereas expression level was lower and similar in BAFF and BAFF-R ko kidneys. In the case of Renin (*Ren1*), expression levels were also similar in BAFF and BAFF-R ko kidneys, but higher in comparison to the wildtype. Kap is expressed in the proximal section of TECs [87] and its function is unknown so far. It seems to be critical for cardiovascular-renal homeostasis, and the overexpression of Kap induces hypertension [88]. Studies showed glomerulosclerosis and proteinuria in transgenic mice [89]. A protective role is discussed as well. It was shown that Cyclosporin A (CsA) induces the downregulation of Kap in the S3 segment of proximal TECs, which led to damage and toxicity [90].

Umod is produced in kidneys by cells of thick ascending limbs and distal tubules. It may act as an inhibitor of calcium crystallization in renal fluids and its excretion in urine may provide defense against urinary tract infections. Umod plays a role in glomerular filtration, kidney development, and organ and tissue specific immune response, and is associated with tubulointerstitial kidney disease, nephritis, and renal tubular atrophy [91,92]. Umod may serve as a receptor for the binding and endocytosis of cytokines and TNF. Studies showed that single nucleotide polymorphisms (SNPs) in the *Umod* gene are associated with GFR and CKD [93,94]. The serum levels of Umod are higher in CKD patients [95]. Nevertheless, protective effects are discussed as well. *Umod* knockout mice, undergoing ischemia/reperfusion (I/R) injury, showed more inflammation, tubular necrosis and a greater impairment of kidney function [96]. Without knowing the precise role of Kap and Umod in our model, we cannot derive the meaning of the expression pattern. Whether the lower expression levels of BAFF and BAFF-R ko are synonymous with enhancement (Kap and Umod negative effects) or worsening (Kap and Umod positive effects), it is not clear. Renin is secreted by kidneys, plays a role for regulation of blood pressure [97] and electrolyte balance and is associated with renal tubular dysgenesis, tubulointerstitial kidney disease and chronic kidney disease [98]. Mutations in the *REN* gene lead to a predisposition to develop AKI [99]. Our results support the need for further analyses on protein level with regard to use of Renin as a prognostic biomarker.

The following table summarizes the analyzed genes and expression pattern with regard to BAFF and BAFF-R ko (Table 1).

In summary, our analyses suggest the involvement of BAFF in CKD, and that besides the BAFF/BAFF-R system even the TWEAK and TWEAK receptor play a role in the chronic phase of GN. With regard to collagen III expression, BAFF ko seems to improve the outcome. From a clinical point of view, this would be an argument in favor of the early use of belimumab in lupus nephritis. Further investigations with larger sample sizes and different time points after the administration of the nephrotoxic serum are needed to verify this hypothesis and learn more about the chronological order of transition from AKI to CKD. In the present study, only female mice were analyzed. In regard to Ougaard et al. [53], no differences in disease progression between sexes were observed for the performed model. In addition, in our previous work, we could not find any evidence on the RNA level that indicated that female hormones influence the results in an acute kidney injury model using BAFF and BAFF-R ko mice [54]. With regard to the detection of *Txnip*, *Gpx3*, *Ccn2*, *Kap*, *Umod* and *Ren1*, further analyses are needed to rule out that detection is not restricted to RNA level and that they are not only upregulated in the chronic phase of kidney injury, so that they can be used as prognostic biomarkers to predict the transition from AKI to CKD.

## 4. Materials and Methods

### 4.1. Ethical Statement

The conducted study, concerning the housing, breeding, handling and experimental procedures, was approved by the local authority (Landesuntersuchungsamt Rheinland-Pfalz, reference number 23 177-07/G 18-1-024), as well as being conducted in accordance with EU Directive 2010/63/EU and the German Animal Welfare Act.

### 4.2. 3R Principle

Each cage was fitted with nesting material and a shelter in the form of a tube. If possible, mice were kept as a minimum in pairs and as far as possible together with littermates. Before starting the experiment, animals were placed in the designated surrounding for at least 7 days to allow an adaptation to the new environment. The habituation to the experimenter was achieved through animal-friendly handling. The necessary fixation procedures were also carefully trained 3–5 days a week. During the trial, in addition to the daily visual inspection, regular trial-specific scoring was carried out by the trial staff in accordance with the available score sheet, including clear instructions for action. In addition, the veterinary service of the University Medical Center was available 24 h a day. If new methods became known in the course of training that could contribute to reducing pain, suffering or harm to animals in experiments, these were implemented in consultation with the animal house management and animal welfare officer if necessary, after official approval.

### 4.3. Laboratory Animals

All three strains used were purchased from The Jackson Laboratory (Bar Harbor, ME, USA). The study was performed with female BAFF KO (B6.129S2-*Tnfsf13b^tm1Msc^*/J, RRID: IMSR_JAX:010572, [100,101]), BAFF-R KO (B6(Cg)-*Tnfrsf13c^tm1Mass^*/J, RRID: IMSR_JAX:007212, [102]) and wildtype (WT), common name B6 (C57BL/6J, RRID: IMSR_JAX:000664, [103]), mice. Housing occurred in a specific-pathogen-free (SPF) unit under standardized conditions (12/12 h light/dark cycle, room temperature 22 ± 2 °C, humidity 50–70%) in individually ventilated cages (IVCs). Food and water were autoclaved and supplied ad libitum. The experimental procedure was carried out with eight-week-old female BAFF KO, BAFF-R KO and WT mice. For this purpose, the mice were housed in a conventional unit under the same standardized conditions (12/12 h light/dark cycle, room temperature 20 ± 2 °C, humidity 50–70%) in filtertop cages.

### 4.4. Experimental Study Design

**Figure 5:**
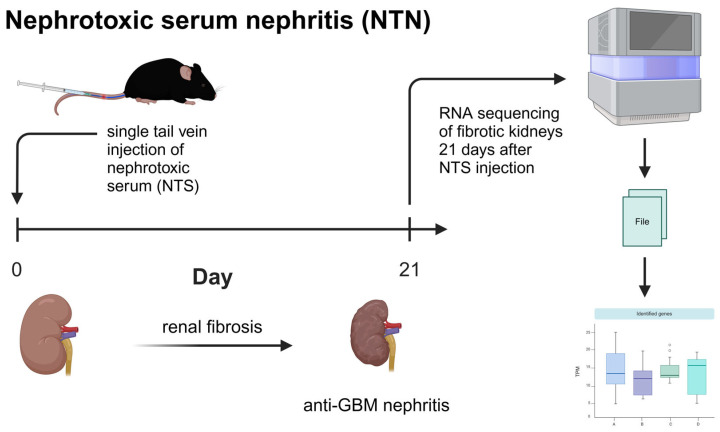
Performed nephrotoxic serum nephritis (NTN) model and bioinformatical analyses. Administration of nephrotoxic serum by single injection into tail vein was performed in BAFF KO (B6.129S2-*Tnfsf13b^tm1Msc^*/J), BAFF-R KO (B6(Cg)-*Tnfrsf13c^tm1Mass^*/J) and wildtype (C57BL/6J) mice. Transcriptome analyses of the kidneys of all three strains were carried out after 21 days. Created with BioRender.com.

In order to investigate the BAFF/BAFF-R system in chronic kidney disease, a nephrotoxic serum nephritis (NTN) model was performed in the BAFF KO (B6.129S2-*Tnfsf13b^tm1Msc^*/J), BAFF-R KO (B6(Cg)-*Tnfrsf13c^tm1Mass^*/J) and wildtype (C57BL/6J) mice (six animals per strain). Then, 21 days after the administration of the nephrotoxic serum, transcriptome analyses of the kidneys were carried out to evaluate differential gene expression (Figure 5).

### 4.5. Nephrotoxic Serum Nephritis (NTN)

Ougaard et al. [53] evaluated the non-accelerated murine nephrotoxic serum nephritis (NTN) model, which was performed in this study. For the induction of passive anti-GBM (glomerular basement membrane) nephritis, a Sheep Anti-Rat Glomerular Basement Membrane (GBM) Serum (Cat. No. PTX-001AGBM, Probetex Inc., San Antonio, TX, USA) was used. Deposits of heterologous IgGs in GBM appear within minutes and the intensity increases the following three to five days. Proteinuria is detected after 24 h. The glomerular localization of autologous IgGs is observed after eight to ten days. Glomerular crescents appear after three weeks and lead to glomerulosclerosis [104]. In our study, the eight-week-old female BAFF KO, BAFF-R KO and WT mice were weighed and fixed in a restrainer (Cat. No. 100680, G&P Kunststofftechnik GbR, Kassel, Germany). The tail was dipped into water at body temperature in order to improve blood circulation so that the veins became pronounced. Before the single administration of nephrotoxic serum (NTS), the tail was disinfected with 70% ethanol. According to GV-SOLAS [105], an allowed maximum volume of 5 µL NTS at body temperature per g body weight were injected into the tail vein. After that, the mice were observed for the following 21 days. Analgesic Buprenorphine (Temgesic, PZN 345928, Indivior, North Chesterfield, VA, USA) was only applied if necessary. Spot urine was collected 21 days after the injection of nephrotoxic serum to determine proteinuria. Median ACR (Albumin Creatinine Ratio) was between 30 and 300 mg/g according to KDIGO [6]. The mice were sacrificed via cervical dislocation, both kidneys were removed, partly shock frozen and stored at −80 °C until RNA sequencing.

### 4.6. RNA Isolation

As described in our previous work [54], shock frozen kidney tissue was initially homogenized in Lysis Solution RL (part of innuPREP RNA Mini Kit, Cat. No. 845-KS-2040250, Analytik Jena GmbH, Jena, Germany) with Tissue Lyser LT (Qiagen N.V., Venlo, The Netherlands). After that, the isolation of total RNA was proceeded with the same kit (details given above) according to the manufacturer’s manual. Finally, the concentration and purity of RNA was determined with NanoDrop 2000 (Thermo Fisher Scientific Inc., Waltham, MA, USA).

### 4.7. RNA Sequencing

The quality check, sequencing and provision of data as FASTQ files were carried out by StarSEQ GmbH (Mainz, Germany) as described in our previous work [54]. An Agilent RNA 6000 Nano Kit (Cat. No. 5067-1511) and Agilent 2100 Bioanalyzer (kit and device from Agilent, Santa Clara, CA, USA) were used for measuring the RNA integrity number (RIN). By using a Qubit RNA High Sensitivity (HS) Assay Kit (Cat. No. Q32855) and Qubit 4 Fluorometer (kit and device from Thermo Fisher Scientific Inc., Waltham, MA, USA), the concentration of RNA was determined. For library preparation, an NEBNext Ultra II Directional RNA Library Prep Kit for Illumina with unique dual index primer pairs (Cat. No. E7760L, New England BioLabs Inc., Ipswich, MA, USA) was used, and the concentration as well as quality were defined with a Qubit 4 Fluorometer by using a Qubit dsDNA HS Assay Kit (Cat. No. Q32854, Thermo Fisher Scientific Inc., MA, USA) and QIAxcel Instrument with QIAxcel ScreenGel 1.5.0 Cartridge (Qiagen N.V., Venlo, Netherlands). The paired-end sequencing of RNA (150 bp read length, approximately 25 million reads per sample (usually more)) was run on Illumina NextSeq 2000 (Illumina, San Diego, CA, USA).

### 4.8. Bioinformatical and Statistical Analyses

Six samples of each strain were investigated in the following bioinformatical analyses. The paired-end RNA Seq data were analyzed on a MacBook with kallisto 0.46.1 [106] using Ensemble Transcriptome v96 *mus musculus* (https://github.com/pachterlab/kallisto-transcriptome-indices/releases, accessed on 13 April 2024) as a reference, manually deleting transcript sequences from hemoglobin chains. The analysis was carried out with R (version 4.1.2) using DeSeq2 [107] for differential gene expression analysis. Based on read counts for each gene in each sample, DeSeq2 generates a generalized linear model of the negative binomial family, followed by several mathematical steps (normalization, estimation of gene-wise dispersion, shrinkage estimation of logarithmic fold changes, Fisher estimation, Wald test and Cook’s distance for outlier detection, adjusting for multiple testing using the procedure of Benjamini and Hochberg [108]). The output of the tool is a log2foldchange as well as an adjusted *p*-value for each gene and thus reflects the variability in gene expression of a particular gene within a group and between groups [107]. Volcano plots were made with the Bioconductor package “Enhanced Volcano” [109]. The figures were produced with ggplot2 [110]. All scripts and gene expression data to reproduce the figures and analysis are available at https://github.com/sebboegel/nts_moeckel_2024 (accessed on 13 April 2024).

## 5. Conclusions

We can conclude that BAFF is involved in CKD, but signaling is not mediated via BAFF-R. Besides the BAFF/BAFF-R system, even the TWEAK/TWEAK receptor axis seems to be involved in autoimmune GN and therefore chronic kidney disease. The identified genes *Txnip*, *Gpx3*, *Ccn2*, *Kap*, *Umod* and *Ren1* may be useful biomarkers and should be further analyzed with regard to the transition from acute to chronic kidney disease. Understanding the processes and interaction of participating networks during the transition from AKI to CKD will be the key to deduce prognostic biomarkers, which will enable the recognition and initiation of therapy before critical, irreversible turnover to CKD.

## Figures and Tables

**Table 1:** Overview of analyzed genes and expression pattern with regard to BAFF and BAFF-R ko.

Gene	Expression	Current Status	References
*Txnip*	lower in BAFF ko BAFF-R ko and wt similar	associated with kidney disease	[59,60,61]
*Gpx3*	higher in BAFF-R ko BAFF ko and wt similar	associated with CKD	[62,63,64,65,66]
*Igfbp7*	higher in BAFF-R ko BAFF ko and wt similar	approved urinary biomarker	[67,68]
*Ccn2*	higher in BAFF-R ko BAFF ko and wt similar	related to renal fibrosis, discussed as fibrotic marker	[69,70,71,72,73,74]
*Kap*	higher in wt BAFF and BAFF-R ko similar	unknown function, expressed in proximal section of TECs	[87,88,89,90]
*Umod*	higher in wt BAFF and BAFF-R ko similar	associated with kidney disease	[91,92,93,94,95,96]
*Ren1*	lower in wt BAFF and BAFF-R ko similar	associated with kidney disease	[97,98,99]

*Txnip*: Thioredoxin Interacting Protein, *Gpx3*: Glutathione Peroxidase 3, *Igfbp7*: Insulin-Like Growth Factor Binding Protein 7, *Ccn2*: Cellular Communication Network Factor 2, *Kap*: Kidney androgen regulated protein, *Umod*: Uromodulin, *Ren1*: Renin. ko: knockout, wt: wildtype.

## Data Availability

All scripts and gene expression data to reproduce the figures and analysis of this paper are available at https://github.com/sebboegel/nts_moeckel_2024 (accessed on 13 April 2024).

## Supplementary Materials

The following supporting information can be downloaded at: https://www.mdpi.com/article/10.3390/ijms25105415/s1.
