# Supplementary material for: Transcriptome Analysis of BAFF/BAFF-R System in Murine Nephrotoxic Serum Nephritis
Source: Int J Mol Sci. 2024 May 16;25(10):5415. doi: 10.3390/ijms25105415 (PMC11121395; doi:10.3390/ijms25105415)
Supplement: Supplementary file 1 [file ijms-25-05415-s001.zip › ijms-2987985-supplementary.pdf]

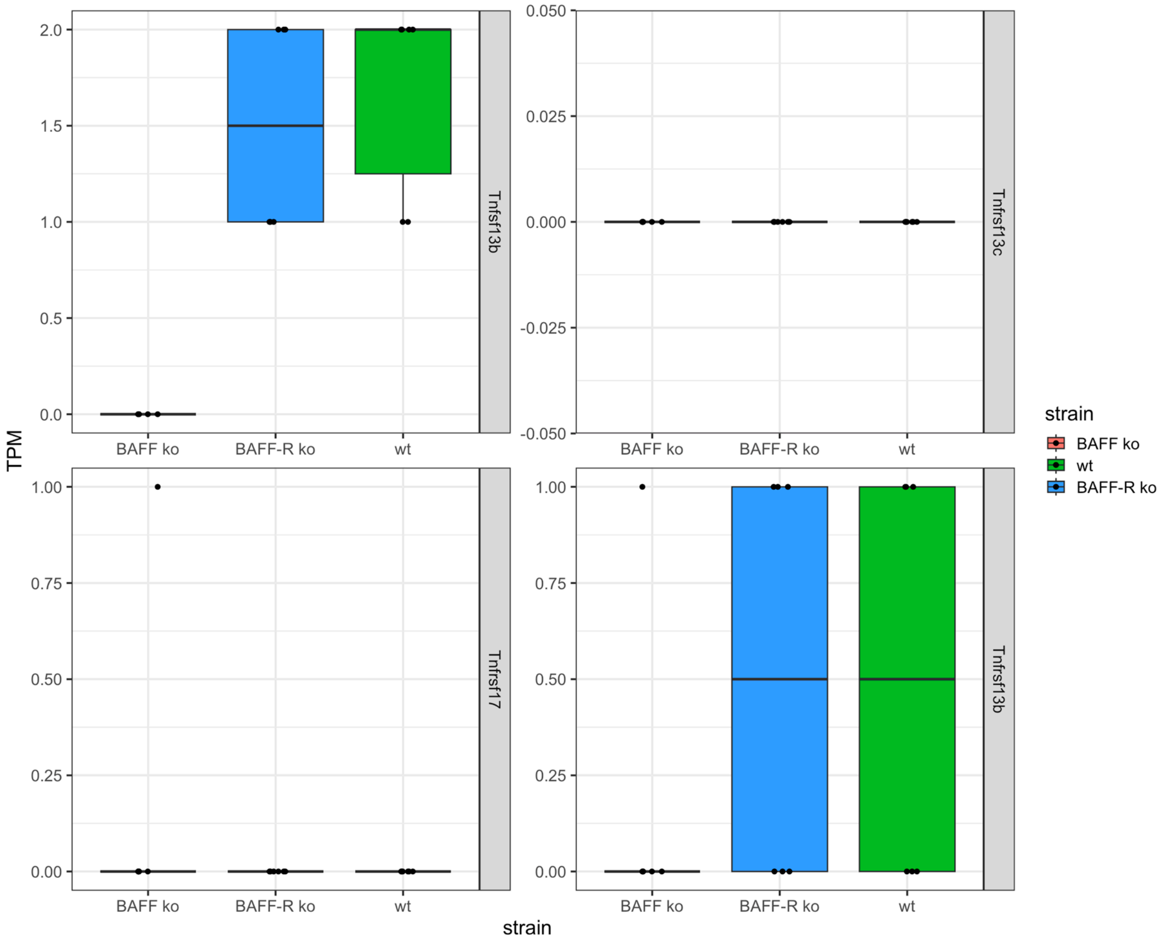

Figure S1: Analyzed genes (BAFF/BAFF-R system) of kidneys 21 days after administration of nephrotoxic serum in BAFF KO (B6.129S2-Tnfsf13b^{tm1Msc}/J), BAFF-R KO (B6(Cg)-Tnfrsf13c^{tm1Mass}/J) and wildtype (C57BL/6J) strain visualized as transcripts per million (TPM). *Tnfsf13b*: BAFF, *Tnfrsf13c*: BAFF-R, *Tnfrsf17*: BCMA, *Tnfrsf13b*: TACI. ko: knockout, wt: wildtype. Each point shows one sample
